# Reduction of eddy current losses in inductive transmission systems with ferrite sheets

**DOI:** 10.1186/s12938-016-0297-4

**Published:** 2017-01-05

**Authors:** Matthias Maaß, Andreas Griessner, Viktor Steixner, Clemens Zierhofer

**Affiliations:** Institute of Mechatronics, University of Innsbruck, Technikerstrasse 13, 6020 Innsbruck, Austria

**Keywords:** Inductive transmission systems, Cochlear implants, Eddy current losses, Ferrite shield, Series–parallel resonant converter

## Abstract

**Background:**

Improvements in eddy current suppression are necessary to meet the demand for increasing miniaturization of inductively driven transmission systems in industrial and biomedical applications. The high magnetic permeability and the simultaneously low electrical conductivity of ferrite materials make them ideal candidates for shielding metallic surfaces. For systems like cochlear implants the transmission of data as well as energy over an inductive link is conducted within a well-defined parameter set. For these systems, the shielding can be of particular importance if the properties of the link can be preserved.

**Results:**

In this work, we investigate the effect of single and double-layered substrates consisting of ferrite and/or copper on the inductance and coupling of planar spiral coils. The examined link systems represent realistic configurations for active implantable systems such as cochlear implants. Experimental measurements are complemented with analytical calculations and finite element simulations, which are in good agreement for all measured parameters. The results are then used to study the transfer efficiency of an inductive link in a series–parallel resonant topology as a function of substrate size, the number of coil turns and coil separation.

**Conclusions:**

We find that ferrite sheets can be used to shield the system from unwanted metallic surfaces and to retain the inductive link parameters of the unperturbed system, particularly its transfer efficiency. The required size of the ferrite plates is comparable to the size of the coils, which makes the setup suitable for practical implementations. Since the sizes and geometries chosen for the studied inductive links are comparable to those of cochlear implants, our conclusions apply in particular to these systems.

## Background

Contactless inductive transmission systems are used in a broad field of applications where wires between transmitter and receiver modules are impractical. The concept is based on inductively coupled coils that allow the simultaneous transfer of power and information. Typical examples can be found, e.g., in non-contact battery chargers for modern cell phones [1] or electric vehicles [2, 3], in the field of robotics [4, 5], RFID systems [6, 7], or wireless sensor networks [8, 9]. In addition, inductive links are used in a variety of biomedical applications for the power and/or information transfer of implanted medical systems, e.g., cochlear implants [10–12].

There is a continuing trend towards miniaturization of industrial and biomedical applications that use inductive transmission systems. This implies that system components with metallic surfaces or metallic interconnections between such components have to be positioned in the vicinity of the coils. If these structures are exposed to the alternating magnetic field of the link, eddy currents are induced due to Faraday’s Law [13]. These eddy currents generate a magnetic field in the opposite direction of the source field resulting in an attenuated overall field. As a result, the coupling factor between the coils is significantly reduced. This, in turn, reduces the power transfer efficiency and diminishes the maximum amount of power that can be transferred.

A fundamental solution to this problem would be to keep metallic objects away from the inductive transmission system, which may not be possible in many cases due to size requirements. In particular, the system studied in this paper is very similar to the inductive transmission system of cochlear implants. Due to the compactness of present devices like single unit processors and prospective even more compact future designs, metallic structures such as the electronics of the speech processor, the microphone or the battery housed in metallic packages can be very close to the transmitter coil, calling for alternative solutions. One possibility is the insertion of a high-permeability ferrite plate between the coil and the conductive structures. The ferrite layer acts like a “magnetic mirror”, which deflects the magnetic flux occurring in the source coil from the conductive layer. This technique provides a shielding of the metallic object associated with a considerable reduction of the induced eddy currents.

For a large variety of systems, the primary goal when using a ferrite layer is the shielding from electromagnetic field interferences [14]. Since the systems we investigate in this paper are similar to already existing medical devices, there are a few different aspects on which we want to focus. One aspect is the transfer efficiency of the link, a property directly linked with the power consumption of the device. Another important aspect is retaining the link parameters like the inductivities and hence the coupling coefficient. Since the mentioned medical devices have well-defined frequencies for energy and data transmission, an unaltered behaviour is advantageous.

**Fig. 1:**
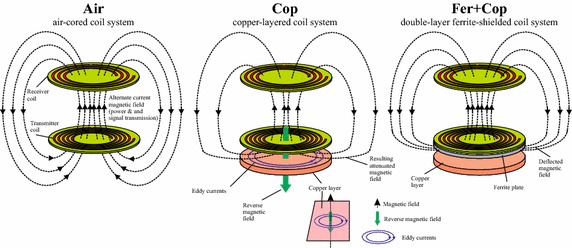
Functional principle of the ferrite shield. The figure shows the investigated coil configurations with the corresponding magnetic *field lines* drawn schematically. *Air* Air-cored coil-system without substrate. *Cop* The single layer of copper substrate represents a detrimental metallic surface where substantial eddy current losses arise. *Fer* *+* *Cop* An additional ferrite layer is used to minimize the eddy current losses

Figure 1 shows the basic shapes of the magnetic fields of magnetically coupled coils for various configurations. In the “air-cored” coil system, the typical undisturbed magnetic field appears. In the “copper-layered” coil system, a copper surface is positioned in the vicinity of the transmitter coil. Eddy currents in the direction shown are induced. The additional magnetic field generated by the eddy currents results in an attenuated overall field. Insertion of a ferrite layer as shown in the “double-layer ferrite-shielded” coil system aims at the deflection of the magnetic field. The magnetic field at the position of the copper layer should be close to zero, and thus almost no eddy currents are induced.

The present paper investigates the suitability of highly permeable ferrite plates for electromagnetic shielding of conductive structures in inductive transmission systems in the context of cochlear implants. We will show that ferrite sheets can be used in this context not only to restore the transfer efficiency of the unperturbed system but also the coil inductivities, coupling strength and consequently the resonance frequencies. A central question is also whether or not the size of ferrite plates for effective shielding can be kept in the same range as the size of the coils. These are prerequisites for already implemented systems to function despite a possible detrimental influence of metallic surfaces close to the link.

The paper is organized as follows. In section “Methods”, we first introduce the analytical model for the inductance calculation of planar spiral windings on single and double-layered substrates. Then, finite element method (FEM) simulations that are used to assess the validity of the analytical approach are presented. In the next subsection, we describe the experimental setup and the parameters used for the different measurement configurations. The behaviour of the inductances and hence the coupling between the coils dependent on various system parameters like substrate radius, number of turns and the distance between the coils is studied in the section “Results and discussion”. In this section the theoretical and FEM predictions are verified by experimental measurements and we also analyze the efficiency of an inductive transmission system in form of a series–parallel resonant converter [12, 15] for different substrate configurations. We summarize our findings in the section “Conclusions”.

## Methods

The goal of the present work is to study the suitability of thin ferrite sheets to shield an inductive link from the unwanted influence of conducting structures close to the link in order to protect the transmission characteristics. Hence, we study the properties of an inductive link on top of three different substrate configurations as shown in Fig. 1:

- *Air*: The original air-cored coil system without substrate serves as a reference.
- *Cop*: The substrate consists of a single layer of copper. This configuration allows the unwanted influence of a conducting material near the link.
- *Fer* *+* *Cop*: The double-layered substrate, with the first layer consisting of ferrite, the second layer of copper. The ferrite sheet is placed between the primary coil and the copper plate and is in direct contact with the conductor in order to keep the magnetic field away from the copper layer (see Fig. 1).

In the following, we will describe and analyze the properties of the different link configurations using an analytical model, FEM simulations and experimental setups.

### Analytical model

For the theoretical description, we have used an analytical model developed by Su et al. [16]. The model is derived under the assumption of an infinite substrate radius and by approximating an *N*-turn spiral coil by a set of *N* circular rings with rectangular cross section. The validity of these assumptions has been confirmed in previous works [16–18].

We will first describe the basics of the model by considering one turn from each of two planar coaxial coils before generalizing to the case of multiple turns for both coils.

**Fig. 2:**
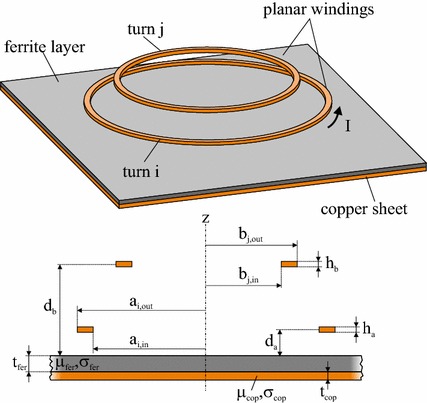
3D and cross-sectional view of two single windings on an infinite double-layer substrate

A three-dimensional and a cross-sectional view of the setup reduced to two turns *i* and *j* in coils *a* and *b* are shown in Fig. 2. The geometry of the two windings is described by the inner and outer radii $a_{{i,{\text{in}}}}$ and $a_{{i,{\text{out}}}}$ ($b_{{j,{\text{in}}}}$ and $b_{{j,{\text{out}}}}$), the thickness *h*_*a*_ (*h*_*b*_) and the distance *d*_*a*_ (*d*_*b*_) above the substrate. The two-layer substrate consists of a ferrite and a copper sheet and is characterized by the relative permeabilities $\mu_{\text{fer}}$ and $\mu_{\text{cop}}$, the electrical conductivities $\sigma_{\text{fer}}$ and $\sigma_{\text{cop}}$, and the layer thicknesses $t_{\text{fer}}$ and $t_{\text{cop}}$.

The turn *i* at *z* = *d*_*a*_ is assumed to carry a harmonic current $I\left( t \right) = I_{0} e^{j\omega t}$ with the angular frequency *ω*. With the mutual impedance *Z*_*ij*_ between the source turn *i* at *z* = *d*_*a*_ and the turn *j* at *z* = *d*_*b*_ we find the induced voltage $V_{ij} = Z_{ij} \cdot I$ [19]. According to [13], the mutual impedance *Z*_*ij*_ can be written as

1 $$Z_{ij} = j\omega M_{ij} + Z_{{{\text{Sub}},ij}} ,$$

where *M*_*ij*_ is the mutual inductance of an air-cored system without the substrate. The contribution to the impedance due to the substrate is denoted by the complex-valued parameter $Z_{{{\text{Sub}},ij}}$. The real part of $Z_{{{\text{Sub}},ij}}$ represents the frequency-dependent eddy current losses in the substrate, the complex part accounts for the increase of the inductance compared with the corresponding air-cored value. Within the validity of the model  [16] the parameters can be calculated as

2 $$M_{ij} = \frac{{\mu_{0} \pi }}{{h_{a} h_{b} \ln \left( {\frac{{a_{{i,{\text{out}}}} }}{{a_{{i,{\text{in}}}} }}} \right)\ln \left( {\frac{{b_{{j,{\text{out}}}} }}{{b_{{j,{\text{in}}}} }}} \right)}}\mathop \int \limits_{0}^{\infty } S\left( {ua_{{i,{\text{out}}}} ,ua_{{i,{\text{in}}}} } \right)S\left( {ub_{{j,{\text{out }}}} ,ub_{{j,{\text{in}}}} } \right)Q\left( {uh_{a} ,uh_{b} } \right)e^{{ - u\left| {d_{b} - d_{a} } \right|}} {\text{d}}u,$$

3 $$Z_{{{\text{Sub}},ij}} = \frac{{j\omega \mu_{0} \pi }}{{h_{a} h_{b} \ln \left( {\frac{{a_{{i,{\text{out}}}} }}{{a_{{i,{\text{in}}}} }}} \right)\ln \left( {\frac{{b_{{j,{\text{out}}}} }}{{b_{{j,{\text{in}}}} }}} \right)}}\mathop \int \limits_{0}^{\infty } S\left( {ua_{{i,{\text{out}}}} ,ua_{{i,{\text{in}}}} } \right)S\left( {ub_{{j,{\text{out}}}} ,ub_{{j,{\text{in}}}} } \right)Q\left( {uh_{a} ,uh_{b} } \right)\lambda \left( {t_{\text{fer}} , t_{\text{cop}} } \right)e^{{ - u\left| {d_{a} + d_{b} } \right|}} {\text{d}}u.$$

The full expressions for *S*, *Q* and *λ* can be found in  [16]. The real valued functions *S* and *Q* depend solely on the geometry of the two turns, whereas the complex-valued parameter function *λ* also depends on the angular frequency *ω*.

Equations (2) and (3) can also be used to calculate the total impedance of a single turn by setting *i* = *j* so that $a_{{i,{\text{out }}}} = b_{{j,{\text{out}}}}$, $a_{{i,{\text{in }}}} = b_{{j,{\text{in}}}}$, *h*_*a*_ = *h*_*b*_ and *d*_*a*_ = *d*_*b*_. Consequently, the exponential term in Eq. (2) disappears and the self-inductance *M*_*i*=*j*_ in air no longer depends on *d*_*a*_. For a detailed description we again refer to  [16].

**Fig. 3:**
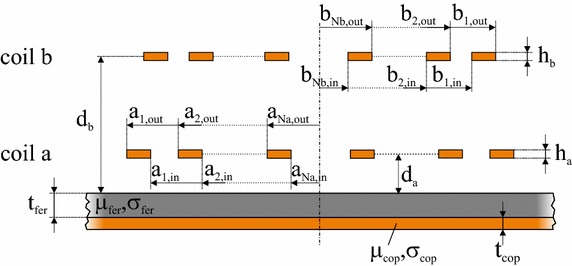
Cross-sectional view of two planar coils on an infinite double-layer substrate

As a next step, we consider the general case of two multiple-turn coils with *N*_*a*_ and *N*_*b*_ turns, respectively, on a double-layer substrate as shown in Fig. 3. The total impedance *Z*_*a*_ of coil *a* can be calculated as the sum of all mutual impedance pairs of coil *a* [20]

4 $$Z_{a} = \mathop \sum \limits_{i = 1}^{{N_{a} }} \mathop \sum \limits_{j = 1}^{{N_{a} }} Z_{ij} = \mathop \sum \limits_{i = 1}^{{N_{a} }} Z_{i = j} \left( {a_{{i,{\text{out}}}} ,a_{{i,{\text{in}}}} } \right) + \mathop \sum \limits_{i \ne j}^{{N_{a} }} Z_{ij} \left( {a_{{i,{\text{out}}}} ,a_{{i,{\text{in}}}} ,a_{{j,{\text{out}}}} ,a_{{j,{\text{in}}}} } \right).$$

The first term on the right hand side of Eq. (4) represents the sum of the impedances *Z*_*i*=*j*_ of all single turns, the second term accounts for the mutual impedances $Z_{ij} \left( {i \ne j} \right)$ between two separate turns. From Eq. (4) we can derive the self-inductance *L*_*a*_ of the coil as

5 $$L_{a} = {\text{Im}}\left( {Z_{a} } \right)/\omega .$$

The calculation for the secondary coil inductance *L*_*b*_ is carried out analogously. The total impedance *Z*_*ab*_ between primary coil *a* and secondary coil *b* is obtained as the sum of all mutual impedances *Z*_*ij*_,

6 $$Z_{ab} = \mathop \sum \limits_{i = 1}^{{N_{a} }} \mathop \sum \limits_{j = 1}^{{N_{b} }} Z_{ij} \left( {a_{{i,{\text{out}}}} ,a_{{i,{\text{in}}}} ,b_{{j,{\text{out}}}} ,b_{{j,{\text{in}}}} } \right).$$

The mutual inductance *M*_*ab*_ can be obtained from the imaginary part of *Z*_*ab*_ as

7 $$M_{ab} = {\text{Im}}\left( {Z_{ab} } \right)/\omega .$$

Having found analytical expressions for *L*_*a*_, *L*_*b*_ and *M*_*ab*_ allows us to compute the coupling coefficient *k* between the two coils of the inductive link via

8 $$k = \frac{{M_{ab} }}{{\sqrt {L_{a} L_{b} } }}.$$

The model for a two-layer substrate can be easily reduced to the simpler case of an inductive link on a single-layer substrate by assuming that the two layers of the substrate are made of the same material. In case of an air-cored inductor (without a substrate), the term *Z*_Sub*,ij*_ in Eq. (1) disappears and the mutual inductance is calculated from Eq. (2).

Equations (2) and (3) have no analytic solution and thus need to be solved numerically. The licensed software package MATLAB R2011b was used for numerical integration. It should be taken into account that all equations of the analytical model were derived under the assumption of ideal boundary conditions, i.e. the radius of the double-layered substrate is infinite. For realistic boundary conditions, the setup was also studied with FEM simulations.

### Finite element simulations

FEM simulations have been performed in ANSYS 11.0. (ANSYS Inc., Canonsburg, US). For the simulations the spiral-shaped coils are replaced with sets of circular windings and rectangular profiles as has been done in the analytical model. This arrangement is rotationally symmetrical and consequently the three-dimensional simulation of the model can be replaced by the analysis of a two-dimensional cross section which fully reflects the 3D setup. With the same number of grid points, the reduction from the 3D to the 2D model allows for a much denser mesh grid at the same computational cost.

An advantage of the ANSYS software is that the simulation geometry can be connected with active and passive discrete components, such as current and voltage sources, resistors and capacitors. In this way it is possible to implement complete inductive transmission networks relatively easily as will be shown in the “Results and discussion” section below.

### Experimental setup

**Fig. 4:**
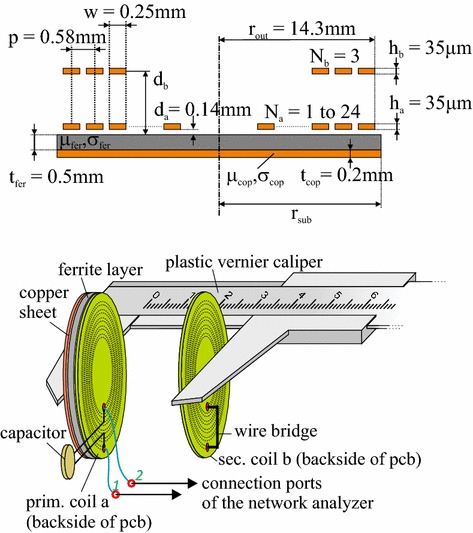
*Top*: Cross-sectional view of the used prototype coil system on a finite double-layer substrate. The number of turns in the secondary coil b is fixed to *N*_*b*_ = 3 while *N*_*a*_ varies between 1 and 24 for different experiments. *Bottom*: Setup for the experimental measurements

In addition to the analytical model and the FEM simulations, the different link configurations were analyzed in experimental setups. In Fig. 4, a schematic setup of the used coil and substrate arrangement is shown as well as the typical geometric dimensions of the realized prototype. The two aligned co-axial and co-planar spiral windings are separated by the axial distance *d* = *d*_*b*_ − *d*_*a*_. The substrate is fixed on top of a thin insulating foil which is placed on the primary coil, so that *d*_*a*_ is fixed to *d*_*a*_ = 0.14 mm, and *d*_*b*_ varies from about 3–10 mm for different setups. This covers the typical coil separations found in real cochlear implant systems. The two coils are identical with respect to the geometric parameters outer radius, pitch, width and thickness. The number of turns is fixed to *N*_*b*_ = *3* for the secondary coil, whereas *N*_*a*_ for the primary coil varies between *1* and *24* in different measurements. The unperturbed air-cored system which serves as a reference operates at 11.7 MHz and has N_a_ = 8 t with an inductance of *L*_0_ = 2.53 µH and an outer coil radius *r*_coil_ = 14.3 mm. The substrate is single or double-layered and consists of disc-shaped ferrite and copper sheets. In case of a double-layered substrate, we use matching substrate radii *r*_sub_, the optimal value for *r*_sub_ will be determined in the subsequent section. The ferrite material used for shielding was IRLG5, a flexible ferrite foil from TDK (*µ*_*r*_ = 50, *σ* = 0.2 S*/*m) with a thickness of 0.5 mm. A 0.2 mm thick copper disc was used to simulate the metallic surface. For the experiments, printed circuit board (PCB) spiral coils were used. The turns are uniformly distributed, i.e. with constant pitch between the turns, starting from the outer coil radius.

**Fig. 5:**
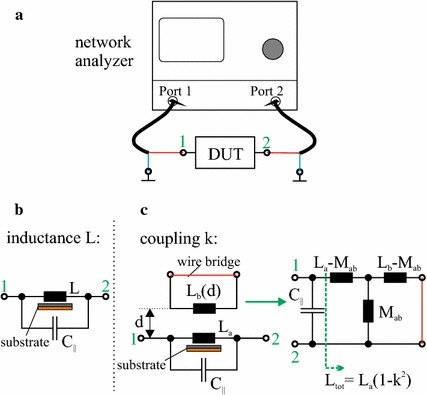
Setup for inductance and coupling measurements. In **a** the connection of the device under test (DUT) to the network analyzer is shown. The specific configurations for measuring the inductance L and the coupling k are shown in **b** and **c**

To study the properties of the different link configurations we use measurements of the self- and mutual inductances and the coupling strength of the link. The setup for the measurements is shown in the bottom plot of Fig. 4. The coils are printed on PCBs with wire connections on the backside of the circuit board with the coil on the front side which limits the minimum coil separation in this setup when a copper or ferrite/copper is present. For the inductance and coupling measurements the two-port device under test is connected between port 1 and port 2 of a vector network analyzer (VNA, here the VNA LA 19-13-02) as shown in Fig. 5a. The measurements are based on the resonance method  [21] which is described in detail in Appendix 1.

## Results and discussion

In this section we will quantify the influence of single or double-layered substrates on the inductive link properties. In the following two subsections the self-inductance of the primary coil is studied as a function of the substrate radius and the number of coils, respectively. We show that a reduction of the number of turns together with the use of a sufficiently large ferrite sheet allows to closely match the inductance of the unperturbed air-cored system. We make use of these findings in the subsequent subsection, where the primary and secondary inductances *L*_*a*_ and *L*_*b*_, the mutual inductance *M*_*ab*_ and the coupling strength *k* are studied as a function of the coil separation *d*, both, for the inductive link on the double-layered substrate (i.e. the interfering conducting material and the ferrite shield) and the original air-cored link. In the last subsection we study the transfer parameters for a transmission system in a practical biomedical implementation.

### Dependence of the primary inductance on the substrate radius

We study the influence of the substrate in the three configurations Air, Cop, Fer + Cop, as described at the beginning of the section “Methods”, on the primary coil inductance *L*_*a*_ as a function of the substrate radius *r*_sub_. The objective of this measurement is to find the minimal value of *r*_sub_ so that the link can still be sufficiently shielded from the perturbing conductor and yields a high level of compliance with the analytical model which is based on an infinite substrate. For reference we here also study a setup with a single-layer ferrite substrate (Fer) which should show a similar behaviour as the combined Fer + Cop setup for larger substrate radii. The number of turns of the primary coil is fixed to N_a_ = 8 in this configuration.

**Fig. 6:**
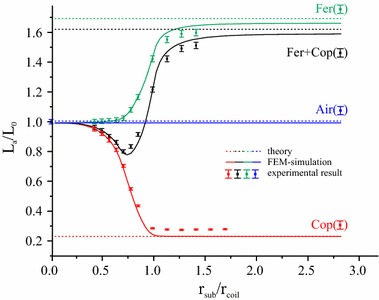
Dependence of the primary inductance *L*_*a*_ on the substrate radius for four substrate configurations obtained from the analytical model, FEM simulations and experimental measurements. *Error bars* indicate the measurement uncertainty due to the limited precision of the instruments

In Fig. 6 the self-inductance of the primary coil is shown as a function of the substrate radius. Analytical (dotted lines), FEM simulation (solid lines) and experimental (markers) results are shown for the four different substrate configurations, error bars in this and the following figures indicate the measurement uncertainty due to the limited precision of the instruments. At the starting point *r*_sub_ = 0 all results approach the value of the air-cored system (shown in blue), except the analytical model which always assumes an infinite substrate. A single layer of copper (red lines and markers) near the link leads to a reduction of the self-inductance with increasing substrate radius, due to the induced eddy currents, which levels off at about *r*_sub_ = *r*_coil_. In contrast, a single-layered ferrite foil (shown in green) results in a significant increase of the initial value. Dependent on the relative permeability and the substrate thickness a theoretical enhancement of up to 100 % compared to the corresponding value of the air-cored system can be obtained  [22]. The ferrite disc acts as a magnetic mirror which leads to an enhancement of the magnetic flux occurring in the coil and thus to an increase of the inductivity. The functionality of the ferrite sheet acting as a shield is also shown schematically in Fig. 1.

The inductance for a double-layered substrate consisting of ferrite and copper (black lines and symbols in Fig. 6) combines the attenuating influence of the copper sheet and the shielding by the ferrite layer. In our setup, the value of the inductance is smaller than *L*_*0*_ for a substrate radius ${\text{r}}_{\text{sub}} \le r_{\text{coil}}$, whereas for values above 0.9 *r*_coil_ the inductance is larger than in the air-cored case. For partly covered coils, the magnetic field is not fully reflected by the ferrite and thus the part of the field which bypasses the substrate induces eddy currents on the back side of the copper disc which results in a reduction of the inductance. An interesting aspect of the interplay of attenuation and enhancement of the inductivity is the resulting formation of an inductance well, in our setup at approximately 0.7 *r*_coil_. The results show a very good agreement between experimental data, FEM simulations and the analytical model predictions, particularly for large substrate radii.

As stated above, the goal of the current measurement is to find a substrate radius which is small enough to enable a compact design and large enough to sufficiently shield the influence of a conductor near the inductive link. From Fig. 6 it can be seen that a possible choice for our configuration is to set *r*_sub_ = 0.9 *r*_coil_, where the influence of the copper and the ferrite cancel each other out. In a real implementation, however, where the exact influence of the conductor near the link is often unknown, it could be difficult to adapt the radius of the ferrite sheet accordingly. A more robust possibility is to use a slightly larger ferrite sheet which reliably shields the influence of a conductor near the link. The original inductance of the primary coil can then be obtained by reducing the number of turns in the coil, as will be shown in the following section. Specifically, in our setup we use a substrate radius of *r*_sub_ = 1.1 *r*_coil_.

For this radius, the inductance depends only weakly on the substrate radius (see Fig. 6) with a value close to the case of a single-layer ferrite substrate. This indicates a robust shielding of the link from the conductor. A further advantage is that with increasing substrate radii, the agreement between the measured and simulated results with the analytical solution is improved.

### Primary inductance as a function of the number of turns

**Fig. 7:**
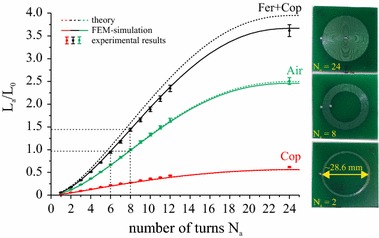
Analytical, FEM and experimental results for the primary inductance *L*_*a*_ are shown as a function of *N*_*a*_ for three substrate configurations. Measurement uncertainties are represented via error bars for the experimental results. Three examples of the used PCB planar spiral coils are shown on the right

The dependence of the primary inductance *L*_*a*_ on the number of turns is studied in the three substrate configurations Air, Cop and Fer + Cop. In accordance with the results of the preceding measurement, the substrate radius was fixed to *r*_sub_ = 1.1*r*_coil_ = 16 mm. The results for the different configurations are shown in Fig. 7. Again, a significant drop in the inductance due to the copper substrate compared to the air-cored system is found, and an increase for the double-layered substrate. We want to design a setup with a double-layered substrate with the goal to closely resemble the link parameters of the initial system. For a coil with *N*_*a*_ = 8 turns, as used in our reference air-cored setup, the value for the Fer + Cop system yields an about 1.5-fold rise in the inductance. The results in Fig. 7 show that this increase can be compensated through a reduction from eight to six turns. Thus, for the subsequent studies the number of turns of the primary coil is fixed to *N*_*a*_ = 6 for all coil configurations utilizing ferrite-layered substrates. Again, all analytical results are in good agreement with FEM simulations and experimentally measured values. As a positive side effect, the reduction of the number of turns leads to a smaller coil resistance. This effect counteracts the ohmic losses which appear in the ferrite foil due to remagnetization processes.

### Influence of the coil separation on the self- and mutual inductances and the coupling coefficient

**Fig. 8:**
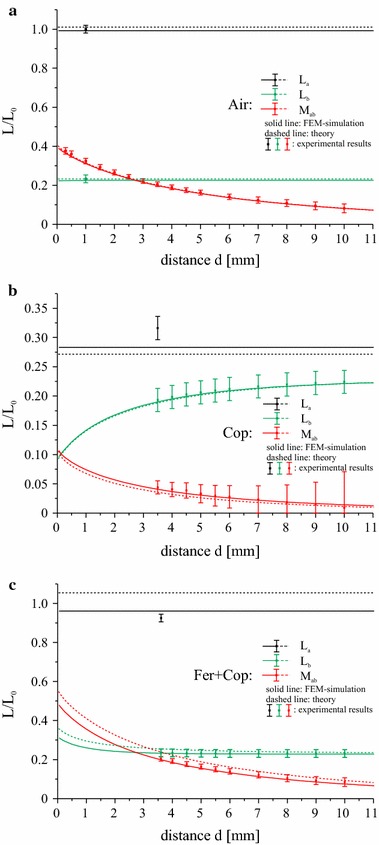
Dependence of the self- and mutual inductances on the coil separation distance *d* for the air-cored coil system (**a**), the copper-layered coil system (**b**) and the double-layer ferrite-shielded coil system (**c**). *Error bars* indicate the measurement uncertainties. Please note the different scale in the *center plot*

So far we have shown that the Fer + Cop setup with r_sub_ = 1.1*r*_coil_ and *N*_*a*_ = 6 turns leads to a primary inductance *L*_*a*_ ≈ 2.53 µH, as in the reference system. The next step is to compare the behaviour of the secondary and mutual inductances *L*_*b*_ and *M*_*ab*_ as well as the coupling coefficient *k* for the fixed parameters as a function of the coil separation *d*. The analysis is again carried out for the three configurations Air, Cop and Fer + Cop. A coil with *N*_*b*_ = 3 turns was selected on the secondary side with an inductance *L*_*b*_ = 0.6 µH. The results for the analytical model, FEM simulations and the measured values are shown in Fig. 8a–c. Note that due to the thickness of the PCB and the wiring in our experimental design (see Fig. 4), the minimal coil separation is limited to about 3.5 mm for the configurations Cop and Fer + Cop. In the air-cored case, the PCB with the primary coil can be flipped and smaller separations can be studied.

For the air-cored system (Fig. 8a), the self-inductances of the primary and the secondary coil are independent of the spacing *d* between the coils. This is why we only show a single value for the respective experimental measurements. In contrast, the mutual inductance *M*_*ab*_ decreases with increasing coil separation (red lines and symbols in Fig. 8a).

The influence of the conducting copper sheet near the link is shown in Fig. 8b. Eddy current losses lead to a strong reduction of both the self-inductances and the mutual inductance. In this setup, the distance between the copper sheet and coil *a* is fixed and the self-inductance *L*_*a*_ is reduced but constant. However, in contrast to the air-cored system, the self-inductance *L*_*b*_ now depends on the distance due to the decreasing influence of the copper sheet on the secondary coil with increasing coil separation. In the limit of large coil separation, *L*_*b*_ approaches the value of the air-cored setup.

As Fig. 8c shows, the insertion of the highly permeable ferrite layer leads to a shielding of the copper disc and compensates very well for the inductance drop. The values of the inductivities are close to the corresponding values of the air-cored system, especially *L*_*a*_ and *M*_*ab*_. The presence of the ferrite sheets leads to a slight increase of the secondary inductance *L*_*b*_ for very small distances.

**Fig. 9:**
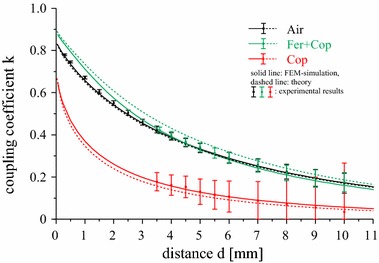
Coupling coefficient *k* as a function of the coil separation distance *d* for three substrate configurations. *Error bars* indicate the measurement uncertainty and are obtained from error propagation calculations

The values of the inductances *L*_*a*_, *L*_*b*_ and *M*_*ab*_ can be used to calculate the coupling coefficient *k* according to Eq. (8). Figure 9 shows the calculated, measured and simulated values of *k* as a function of the coil separation *d* for the three substrate configurations. As for the inductances, the influence of the copper sheet leads to a strong drop in the coupling strength (red lines and symbols). The very good agreement of the results for the double-layered substrate (green) with the reference air-cored setup (black) confirms that the ferrite layer provides an adequate shielding of the copper sheet and that the original system can be reproduced very well with the chosen substrate radius and the reduction in the number of turns.

Figures 8a–c and 9 show that the results of the FEM simulations coincide very well with the experimental measurements, the small deviations of the analytical values are due to the assumption of infinite substrate radii. This shows that both the FEM simulations and the assumed model  [16] can be used as an efficient tool to reliably predict the properties of an inductive link with a variety of substrate configurations.

### Transfer parameters for a transmission system in a practical implementation

In the preceding sections we have shown that a ferrite shield is capable of countervailing the reduction in inductances and coupling strength caused by a metallic surface close to an inductive link. In the following we will analyze a practical implementation of a complete inductive transmission system as used in real systems like cochlea implants. We will show that the use of a ferrite shield does not significantly deteriorate the characteristic transfer parameters for practical implementations of the complete inductive transmission system compared to an equivalent air-cored coil system.

**Fig. 10:**
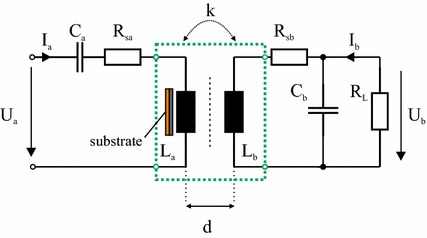
Circuit diagram of the inductive transmission system in series–parallel resonant topology

**Fig. 11:**
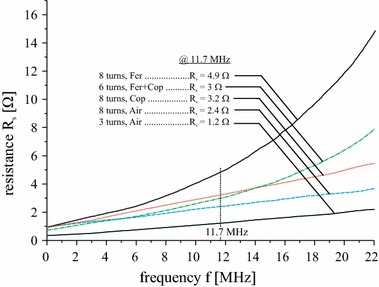
Series resistance *R*_*s*_ of planar coils on finite substrates as a function of the operating frequency *f*

Our implementation of the inductive transmission system has a series–parallel topology as shown in Fig. 10. It consists of a series resonant circuit on the primary side and a parallel resonant circuit on the secondary side at an operating frequency of 11.7 MHz. The elements *C*_*a*_ and *L*_*a*_ characterize the series resonant circuit, *C*_*b*_ and *L*_*b*_ form the parallel resonant circuit. The resistance *R*_*L*_ is the combination of the occurring loads. In our setup we used *C*_*a*_ = 81 pF, *C*_*b*_ = 275 pF and *R*_*L*_ = 620 Ω which are parameters typically used in the field of cochlea implants. The coils themselves are approximated by their equivalent series circuits, where *R*_*sa*_ and *R*_*sb*_ characterize the series resistances of primary and receiver coils, respectively. The values of the series resistors of the coils are frequency-dependent and their values *R*_*sa*_(*f*) and *R*_*sb*_(*f*) are shown in Fig. 11.

For the calculation of the power transfer efficiency *η* and the voltage gain *ν*, the inductive link is modelled by its T-equivalent circuit, as shown in Fig. 5c. From the Z-parameter representation of the complete network we obtain

9 $$\eta = \frac{{\left| {I_{b} } \right|^{2} R_{L} }}{{{\text{Re}}\left\{ {U_{a} I_{a}^{*} } \right\}}} = \frac{{R_{L} }}{{{\text{Re}}\left\{ {\left( {z_{11} \frac{{R_{L} + z_{22} }}{{z_{21} }} - z_{12} } \right)\left( {\frac{{R_{L} + z_{22} }}{{z_{21} }}} \right)^{*} } \right\}}}$$

10 $$\nu = \left| {\frac{{U_{b} }}{{U_{a} }}} \right| = \frac{{R_{L} }}{{\left| {z_{11} \frac{{R_{L} + z_{22} }}{{z_{21} }} - z_{12} } \right|}}.$$

The expressions for the Z-parameters are given in Appendix 2.

**Fig. 12:**
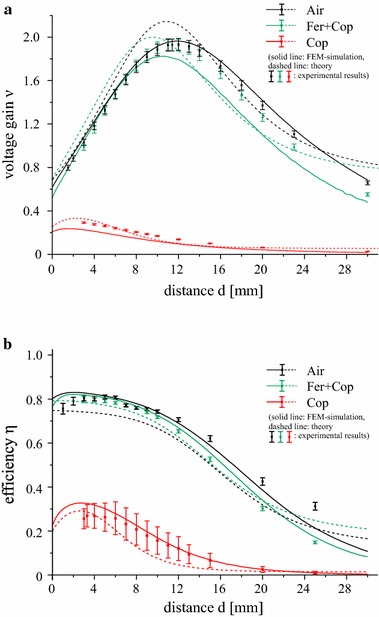
**a** Voltage gain *v* and **b** power transfer efficiency *η* as a function of the separation distance *d* at 11.7 MHz for three substrate configurations. *Error bars* indicating measurement uncertainties are shown for the experimental results

The values from the analytical model, the FEM simulations and the experimental measurements have been used to calculate the voltage gain and the power transfer efficiency via Eqs. (9) and (10). In Fig. 12 we show the results for ν and *η* as a function of the coil separation *d.* The significant drop in both transfer parameters ν and *η* for the case of a copper substrate (red) shows the importance of an efficient shielding against an unwanted influence of conducting media close to an inductive link. The results for the double-layer substrate configuration Fer + Cop (green lines and markers) are very close to the respective results of the air-cored reference setup. This proves the capability of the ferrite foils to restore the efficiency of an inductive transmission system. Again, the analytical results are in good agreement with those obtained by experiment and FEM simulation, especially for small coil separations. As can be seen from Fig. 11, the series resistance of the air-cored setup with *N*_*a*_ = 8 is close to the value for a copper substrate and *N*_*a*_ = 8 at the operating frequency of 11.7 MHz. Thus, the significant deterioration of the transfer characteristics of the inductive transmission system cannot be explained by the drop of the resistance alone but suggests that it is mainly caused by the drop in coupling due to the copper sheet. As the results demonstrate, this shortcoming can be eliminated almost completely by a ferrite shield.

## Conclusions

In this paper we have analyzed the suitability of ferrite sheets as a shield of inductive transmission systems against the influence of metallic surfaces. In particular, we investigated the influence of single or double-layered substrates made of ferrite and/or copper on the link parameters, such as self-inductance and coupling. All results were obtained theoretically with a detailed analytical model as well as with FEM simulations and in addition were confirmed experimentally. The measurements show that copper-covered coils in inductive link systems lead to a significant drop of the self-inductance and coupling due to induced eddy current losses. We could demonstrate that a high shielding performance can be achieved by placing a finite ferrite plate between the coil and the copper disc. In this way, a setup with the same coil configuration leads to even higher values of the primary inductance for the double-layer configuration than for the air-cored reference system. The original link parameters such as inductances and coupling and hence the transfer efficiency can be retained through a reduction in the number of turns. Furthermore, we have shown that the shielding can be achieved for relatively small radii of the ferrite foil in the same order of magnitude as the coil radius which makes them a promising candidate for practical implementations. Our results are particularly relevant for cochlear implant systems, since coil dimensions in these systems closely match the ones we used in our examinations.

As a next step we investigated the transfer parameters for a transmission system in a practical biomedical implementation, where the coils are parts of a transmitter and receiver resonant circuit. In this case, our analysis shows that a copper substrate mimicking a detrimental conductor near an inductive transmission system leads to a massive drop in power transfer efficiency and voltage gain. The results demonstrate that the ferrite layer ensures excellent shielding from the unwanted influence of the copper sheet. The obtained values for the power transfer efficiency *η* and for the voltage link gain *ν* are comparable to the values of the air-cored coil system. In all our investigations we found the measurements to be in good agreement with the calculations based on the analytical model and with FEM simulations. This good agreement shows that simulations and analytical calculations are a good possibility to describe transmission systems on various substrate configurations without having to carry out the sometimes cumbersome experiments.

## Appendix 1

For the measurement of an inductance *L*, a series-through configuration as shown in Fig. 5b is implemented. A known test capacity *C*|| is connected in parallel to the coil. The resonance frequency *f*_0_ of this parallel LC circuit is given by

11 $$f_{0} = \frac{1}{{2\pi \sqrt {L\left( {C_{L} + C_{\parallel } } \right)} }},$$

where *C*_*L*_ is the self-capacitance of the coil. The resonance frequency is determined from the measurement data of the forward transmission parameter *S*_12_ via the VNA. As a next step the measurement is repeated without *C*|| to obtain the self-resonance frequency *f*_*s*_ of the coil which, together with *f*_0_, can be used to calculate the self-capacitance of the coil

$$C_{L} = C_{\parallel } \frac{{f_{0}^{2} }}{{f_{s}^{2} - f_{0}^{2} }} .$$

Rearranging (11) and eliminating *f*_*s*_ yields the self-inductance

$$L = \frac{1}{{\left( {2\pi f_{0} } \right)^{2} \left( {C_{L} + C_{\parallel } } \right)}}.$$

Similarly, the resonance method can be used for the determination of the coupling coefficient *k*. For this, the measurement capacity is switched parallel to the primary coil *L*_*a*_ and the secondary coil *L*_*b*_ is short circuited by a wire bridge as shown in Fig. 5c. Due to the presence of the substrate, the secondary inductance *L*_*b*_ depends on the separation distance *d*. The simplification of the inductively coupled coil system is accomplished by the introduction of the T-equivalent circuit of the two coupled coils. Knowing $M_{ab} = k\sqrt {L_{a} L_{b} }$, it can be shown that the inductive network can be replaced by an equivalent total inductance *L*_*tot*_

12 $$L_{\text{tot}} = L_{a} \sqrt {1 - k^{2} } .$$

The total inductance *L*_tot_, together with *C*||, again forms a parallel resonant circuit with the resonance frequency *f*_*0*_ as given in Eq. (11) now with the inductance *L*_tot_. Together with (12) we find

$$k = \sqrt {1 - \frac{1}{{\left( {2\pi f_{0} } \right)^{2} \left( {C_{L} + C_{\parallel } } \right)L_{a} }}} ,$$

where the values of L_a_ and C_L_ are known from the previous inductance measurements.

## Appendix 2

The Z-parameters can be calculated from the T-equivalent circuit as shown in Fig. 5c. After some algebraic rearrangements we find

$$\begin{aligned} z_{11} & = \left. {\frac{{U_{a} }}{{I_{a} }}} \right|_{{I_{b} = 0}} = R_{sa} + \frac{1}{{j\omega C_{a} }} + j\omega L_{a} + \frac{{jC_{b} M_{ab}^{2} \omega^{3} }}{{1 - \omega^{2} L_{b} C_{b} + j\omega R_{sb} C_{b} }}, \\ z_{12} & = \left. {\frac{{U_{a} }}{{I_{b} }}} \right|_{{I_{a} = 0}} = \frac{{j\omega M_{ab} }}{{1 - \omega^{2} L_{b} C_{b} + j\omega R_{sb} C_{b} }}, \\ z_{21} & = \left. {\frac{{U_{b} }}{{I_{a} }}} \right|_{{I_{b} = 0}} = z_{12} , \\ z_{22} & = \left. {\frac{{U_{b} }}{{I_{b} }}} \right|_{{I_{a} = 0}} = \frac{{R_{sb} + j\omega L_{b} }}{{1 - \omega^{2} L_{b} C_{b} + j\omega R_{sb} C_{b} }}. \\ \end{aligned}$$

## Abbreviations

CI: cochlear implant
DUT: device under test
FEM: finite element method
PCB: printed circuit board
RFID: radio frequency identification
VNA: vector network analyzer
